# Transport of degradable contaminants in a composite vertical cut-off wall under nonlinear adsorption conditions

**DOI:** 10.1038/s41598-025-26086-x

**Published:** 2025-11-26

**Authors:** Hai Lin, Shiyue Ouyang, Jinsong Huang, Qingzhu Li

**Affiliations:** 1 Engineering Research Center of Watershed Carbon Neutralization, Ministry of Education, Nanchang University, 999 Xuefu Avenue, Honggutan District, Nanchang, 330031 Jiangxi Province China; 2 School of Infrastructure Engineering, Nanchang University, 999 Xuefu Avenue, Honggutan District, Nanchang, 330031 Jiangxi Province China; 3 Discipline of Civil, Surveying and Environmental Engineering, The University of Newcastle, Callaghan, NSW 2308 Australia; 4 School of Metallurgy and Environment, Central South University, Changsha, 410083 China

**Keywords:** Cut-off wall, Degradable contaminants, Freundlich adsorption, Transport law, Breakthrough time, GCL, Engineering, Environmental sciences, Hydrology

## Abstract

Barrier materials frequently exhibit nonlinear adsorption characteristics for contaminants in leachate, with this nonlinearity becoming increasingly pronounced at higher concentrations. A composite vertical cut-off wall, constructed from an adsorptive soil-bentonite mixture and a geosynthetic clay liner provides an effective solution for barrier systems and emergency responses to organic contaminants. Understanding the transport mechanisms of degradable contaminants (e.g., organic pollutants) within the composite cut-off wall is essential for evaluating barrier effectiveness against contamination. This study established a general theoretical model for the one-dimensional transport of degradable contaminants in the composite vertical cut-off wall, considering nonlinear adsorption, advection, diffusion, mechanical dispersion, and degradation processes during the transport period simultaneously. Parametric studies show that the relative concentrations of degradable contaminants in the composite cut-off wall, based on the Freundlich adsorption assumption, significantly differ from those predicted by the linear adsorption assumption. The influence of the Freundlich adsorption model parameters on contaminant transport and boundary conditions was thoroughly analyzed. The degradation process can effectively reduce the relative concentration of contaminants in the composite cut-off wall. Notably, the time required for the contaminant concentration to traverse the composite cut-off wall and reach the limit value (breakthrough time), when considering degradation (*t*_1/2_ = 10 a), is 1.7 times that calculated without accounting for degradation. Furthermore, increased permeability of the aquifer under the boundary conditions results in lower contaminant concentrations within the outlet range of the transport model, leading to a corresponding increase in breakthrough time.

## Introduction

Industrial wastewater and leachate from landfills often contain elevated concentrations of degradable contaminants (e.g., organics). These contaminants can infiltrate barrier systems, such as bentonite liners, during seepage, resulting in the contamination of surrounding strata and groundwater^1,2^. Understanding the transport dynamics of degradable contaminants in barrier systems is crucial for assessing and developing impermeable barrier technologies at contamination sources. Vertical cut-off walls are particularly effective in retarding the movement of contaminants from these sources to adjacent areas^3–5^. As a result, this technology has experienced rapid advancements in recent years and has been implemented in the in-situ closure treatment and emergency disposal of landfills. Recently, a novel composite vertical cut-off wall composed of an adsorptive soil-bentonite mixture and a geosynthetic clay liner (GCL) has garnered attention due to its low permeability, ease of construction, and efficacy against organic contaminants^6,7^. The GCL is positioned against one or both sidewalls of the excavated trench, followed by backfilling with an adsorptive soil-bentonite mixture to create a highly integrated composite barrier. Understanding the transport mechanisms of degradable contaminants within the composite vertical cut-off wall is essential for evaluating and improving contamination source control technologies in geo-environmental engineering.

The transport process of contaminants within vertical cut-off walls is influenced by advection, diffusion, and adsorption^8–11^. Analytical solutions and numerical methods are continually being refined to quantitatively elucidate the transport dynamics of contaminants in strata or barrier systems^12–14^. Analytical solutions, in particular, offer a clear representation of how various factors influence the transport mechanisms of contaminants, providing superior computational efficiency compared to numerical simulation methods^15^. The outlet boundary conditions for the model can be tailored to specific application scenarios, including Dirichlet-type boundary^16^, Neumann-type boundary^17^, semi-infinite boundary^18,19^, and Robin-type boundary^20^. Presently, the effects of advection, diffusion, and adsorption can be integrated into the analytical solutions for contaminant transport^21–23^, with one-dimensional transient transport models being the mainstream. Peng et al. ^24^ introduced an analytical solution for the two-dimensional transient transport of organic contaminants in vertical cut-off walls with geomembrane, highlighting diffusion as the primary transport mechanism. For complex contaminant transport scenarios where analytical solutions prove unattainable, numerical methods such as the finite element method and boundary element method can be employed, depending on specific engineering contexts to address the challenges presented^25–28^. Although simulation modeling and iterative calculations often demand significant effort and time, unverified computational results can easily lead to skepticism.

The adsorption process profoundly impacts contaminant transport^29,30^, making the adsorption assumptions applied to contaminants in analytical solution models critical for the accuracy of calculated results^31^. Nevertheless, most existing transport models simplify the adsorption process by adopting a linear adsorption relationship, requiring only a single parameter: the distribution coefficient (*K*_*d*_). Extensive studies^32–34^ have shown that engineering barrier materials, including soil-bentonite mixtures and modified soil-bentonite mixtures utilized in constructing cut-off walls, often exhibit pronounced nonlinear characteristics in their adsorption curves (fitted to either the Freundlich or Langmuir isotherm) for heavy metals and organic contaminants. These nonlinear characteristics are particularly evident under conditions of high contaminant concentration. Consequently, the linear adsorption assumption neglects the actual adsorption behaviors of strata while facilitating model solutions. Thus, it is imperative to develop solutions for contaminant transport under nonlinear adsorption conditions to accurately represent the transport dynamics of contaminants and assess the barrier efficacy of vertical cut-off walls. However, deriving analytical solutions for contaminant transport models that account for nonlinear adsorption remains a significant challenge. Jiang et al.^35^ modeled the transport process considering the nonlinear adsorption of heavy metal ion contaminants in a composite liner, obtaining numerical solutions for the transport dynamics through the finite difference method due to the inability to solve the corresponding mathematical differential equations analytically. This work serves as a reference for addressing the nonlinear adsorption assumption in contaminant transport calculations.

Organic contaminants in leachate degrade over time^36–38^, significantly influencing contaminant transport in barrier systems^39,40^. Additionally, the nonlinear adsorption characteristics of contaminants within the barrier must be considered, given the relatively high concentrations of these contaminants in leachates. The transport of degradable contaminants in the composite vertical cut-off wall entails the interplay of convection, diffusion, nonlinear adsorption, and degradation. Existing research on analytical solutions for contaminant transport has yet to comprehensively address the effects of these factors simultaneously. To effectively evaluate the barrier performance of the composite vertical cut-off wall against degradable contaminants in real-world engineering scenarios, this study establishes a one-dimensional transport model that incorporates the effects of nonlinear adsorption, advection, diffusion, mechanical dispersion and degradation. The aim is to provide a new reference for assessing the service performance of the composite cut-off wall while enhancing the understanding of contaminant transport within the field of geo-environmental engineering.

## Transport model of degradable contaminants in the composite vertical cut-off wall

### Theoretical transport model and fundamental assumptions

**Fig. 1:**
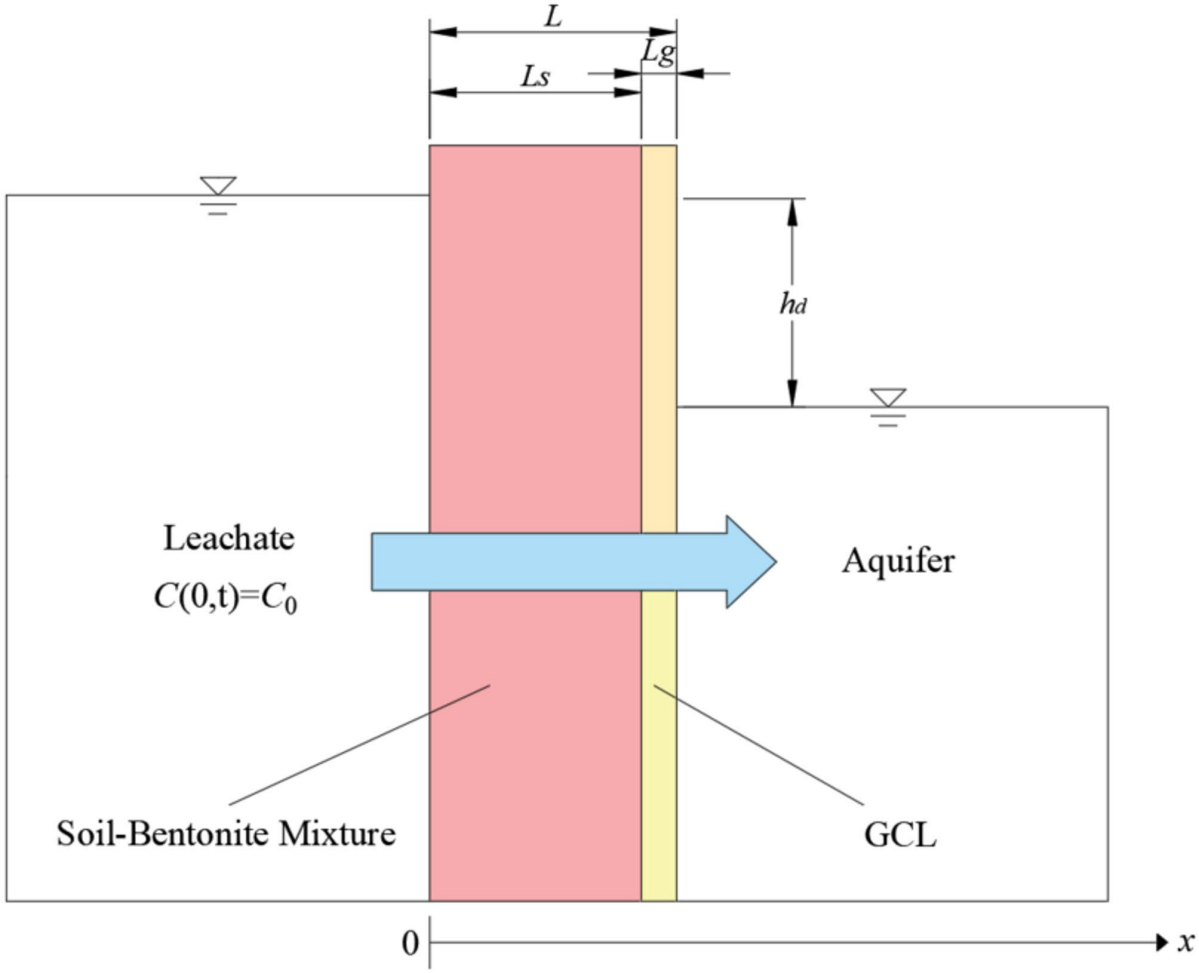
Schematic diagram illustrating the one-dimensional transport model of degradable contaminants within the composite vertical cut-off wall.

The schematic diagram of the one-dimensional transport model for degradable contaminants in the composite vertical cut-off wall is shown in Fig. 1. For clarity, a one-dimensional Cartesian coordinate system is established horizontally to the right at the upstream interface of the composite cut-off wall, denoted by *x*. The thicknesses of the soil-bentonite mixture, GCL, and the entire composite vertical cut-off wall are denoted as *L*_*s*_, *L*_*g*_, and *L*, respectively. The hydraulic head difference between the two sides of the composite cut-off wall is *h*_*d*_, and the concentration of degradable contaminants in the upstream leachate is set to a constant value *C*_0_ for simplicity. An aquifer layer with certain permeability and diffusivity lies to the right of the composite cut-off wall.

To formulate the mathematical expression for the theoretical transport model and facilitate model resolution, the following fundamental assumptions are established, referencing previous theoretical foundations^40–43^:

(1) The soil-bentonite mixture layer and GCL are assumed to be homogeneous, isotropic, and saturated;

(2) Liquid-phase seepage occurs in a steady state and adheres to Darcy’s law;

(3) Contaminant transport involves multiple processes, including advection, diffusion, mechanical dispersion, adsorption, and degradation;

(4) Contaminant diffusion complies with Fick’s second law;

(5) The adsorption of contaminants onto the soil-bentonite mixture layer and GCL is represented by the nonlinear isothermal Freundlich model in equilibrium;

(6) The degradation process in the composite vertical cut-off wall is described by a first-order degradation model.

### Governing equation

Based on the research by Rowe and Brachman^44^, the governing equation for degradable contaminant transport in the composite vertical cut-off wall can be expressed as Eq. (1), which integrates the processes of advection, diffusion, mechanical dispersion, adsorption, and degradation.

1 $$\frac{{\partial {C_s}\left( {x,t} \right)}}{{\partial t}}=\frac{{{D_s}}}{{{R_{F,s}}}}\frac{{{\partial ^2}{C_s}\left( {x,t} \right)}}{{\partial {x^2}}} - \frac{{{v_s}}}{{{R_{F,s}}}}\frac{{\partial {C_s}\left( {x,t} \right)}}{{\partial x}} - \lambda {C_s}\left( {x,t} \right) (0 \leqslant x \leqslant {L_s})$$

In this equation, *C*_*s*_(*x*, *t*) denotes the degradable contaminant concentration in the soil-bentonite mixture layer at time *t* and position *x*. The hydrodynamic dispersion coefficient in the soil-bentonite mixture layer is represented by *D*_*s*_, and *v*_*s*_ denotes the seepage flow velocity in the soil-bentonite mixture layer. The retardation factor in the soil-bentonite mixture layer is represented by *R*_*F*,*s*_, while *λ* is the degradation rate coefficient in this layer. The parameters *R*_*F*,*s*_ and *λ* can be determined using Eqs. (2) and (3), respectively.

2 $${R_{F,s}}=1+\frac{{{\rho _{d,s}}}}{{{n_s}}}{K_{F,s}}{N_s}{C^{{N_s} - 1}}$$

3 $$\lambda =\frac{{\ln 2}}{{{t_{1/2}}}}$$

In these equations, *n*_*s*_ represents the porosity of the soil-bentonite mixture layer, and *ρ*_*d*,*s*_ denotes the dry density of the soil-bentonite mixture layer. *K*_*F*,*s*_ and *N*_*s*_ are the Freundlich adsorption constant and the Freundlich empirical constant of contaminants in the soil-bentonite mixture layer, respectively, while *t*_1/2_ is the degradation half-life of degradable contaminants in the soil-bentonite mixture layer.

Considering advection, diffusion, mechanical dispersion, adsorption, and degradation, the governing equation for contaminant transport in GCL can be expressed as

4 $$\frac{{\partial {C_g}\left( {x,t} \right)}}{{\partial t}}=\frac{{{D_g}}}{{{R_{F,g}}}}\frac{{{\partial ^2}{C_g}\left( {x,t} \right)}}{{\partial {x^2}}} - \frac{{{v_g}}}{{{R_{F,g}}}}\frac{{\partial {C_g}\left( {x,t} \right)}}{{\partial x}} - \lambda {C_g}\left( {x,t} \right) ({L_s} \leqslant x \leqslant L)$$

where *C*_*g*_(*x*,*t*) denotes the degradable contaminant concentration in the GCL at time *t* and position *x*, *D*_*g*_ is the hydrodynamic dispersion coefficient in GCL, *v*_*g*_ is the seepage flow velocity in GCL, and *R*_*F*,*g*_ denotes the retardation factor in GCL. The parameter *R*_*F*,*g*_ can be obtained by Eq. (5).

5 $${R_{F,g}}=1+\frac{{{\rho _{d,g}}}}{{{n_g}}}{K_{F,g}}{N_g}{C^{{N_g} - 1}}$$

where *n*_*g*_ is the porosity of GCL, *ρ*_*d*,*g*_ denotes the dry density of GCL, *K*_*F*,*g*_ and *N*_*g*_ are the Freundlich adsorption constant and the Freundlich empirical constant of contaminants in GCL. The hydrodynamic dispersion coefficient can be expressed as the sum of the effective diffusion coefficient and the corresponding mechanical dispersion coefficient^45^. Therefore, *D*_*s*_ and *D*_*g*_ are obtained by Eqs. (6) and (7).

6 $$\left\{ \begin{gathered} {D_s}={D_s}^{*}+{D_{m,s}} \hfill \\ {D_g}={D_g}^{*}+{D_{m,g}} \hfill \\ \end{gathered} \right.$$

7 $$\left\{ \begin{gathered} {D_{m,s}}={\alpha _{L,s}}{v_s} \hfill \\ {D_{m,g}}={\alpha _{L,g}}{v_g} \hfill \\ \end{gathered} \right.$$

where *D*_s_^*^ and *D*_g_^*^ denote the effective diffusion coefficients in the soil-bentonite mixture layer and GCL, respectively; *D*_*m*,*s*_ and *D*_*m*,*g*_ are the mechanical dispersion coefficients in the soil-bentonite mixture layer and GCL, respectively; *α*_*L*,*s*_ and *α*_*L*,*g*_ are the longitudinal dispersivities in the soil-bentonite mixture layer and GCL, respectively.

### Initial and boundary conditions

The background concentration of the degradable contaminant in the composite vertical cut-off wall is zero at the initial time, and the initial condition in Eq. (8) can be established. The contaminant source concentration at the left boundary of the composite vertical cut-off wall is assumed to be a constant *C*_0_ (mg/L), which can be expressed as Eq. (9).

8 $${C_s}\left( {x,0} \right)={C_g}\left( {x,0} \right)=0 (0 \leqslant x \leqslant L)$$

9 $${C_s}\left( {0,t} \right)={C_0} \left( {x=0,t>0} \right)$$

At the interface between the soil-bentonite mixture layer and GCL, the continuity conditions of concentration and transport flux need to be satisfied [i.e., Eqs. (10) and (11)].

10 $${C_s}\left( {{L_s},t} \right)={C_g}\left( {{L_s},t} \right)$$

11 $${n_s}{v_s}{C_s}\left( {{L_s},t} \right) - {n_s}{D_s}\frac{{\partial {C_s}\left( {{L_s},t} \right)}}{{\partial x}}={n_g}{v_g}{C_g}\left( {{L_s},t} \right) - {n_g}{D_g}\frac{{\partial {C_g}\left( {{L_s},t} \right)}}{{\partial x}}$$

For the right outlet boundary of GCL, it is assumed to be an aquifer layer with certain permeability and diffusivity. Existing studies^18,46^often use different boundary conditions according to different outlet circumstances. When the outlet boundary is an aquifer layer, the Dirichlet-type boundary is commonly used, meaning the outlet boundary concentration is zero. When the outlet boundary is an aquitard layer, the Neumann-type boundary is often used, indicating that the outlet boundary gradient is zero. Considering the differences in diffusivity and permeability of the outlet boundary conditions under various circumstances, the aquifer layer may not be completely permeable. The Robin-type boundary is used for analysis and discussion in this paper, which applies to situations where the outlet boundary has certain diffusivity and permeability^17,47^. The specific expression for the Robin-type boundary is provided in Eq. (12).

12 $$\frac{{\partial {C_g}(L,t)}}{{\partial x}}= - \mu {C_g}(L,t)$$

where *µ* is the Robin-type boundary correlation constant greater than 0, with the unit of *µ* being m^− 1^. The value of *µ* is related to the diffusivity and permeability of the aquifer layer. When *µ* = +∞ m^− 1^, the Robin-type boundary degenerates into the Dirichlet-type boundary. When *µ* = 0 m^− 1^, the Robin-type boundary degenerates into the Neumann-type boundary.

## Numerical solution of the theoretical transport model

Given that the adsorption retardation factors (*R*_*F*_) in the governing equations of the proposed transport model vary with thickness or time, developing analytical solutions for the partial differential equations with variable coefficients is challenging. In this study, the finite difference method is adopted to obtain the numerical solutions of the mathematical governing equation due to its strong applicability and high calculation accuracy^48^.

The thickness of the GCL is much less than that of the soil-bentonite mixture layer. Two different spatial steps are proposed in this study. It is assumed that Δ*x*_1_ and Δ*x*_2_ are the spatial steps of the soil-bentonite mixture layer and GCL, respectively. By dividing the space equally, the corresponding spatial interval numbers for the soil-bentonite mixture layer and GCL are *I*_1_ = *L*_*s*_/Δ*x*_1_ and *I*_2_ = *L*_*g*_/Δ*x*_2_, respectively. Similarly, the time step is denoted as Δ*t*, and the time interval numbers for a given time *t*_0_ are *J* = *t*_0_/Δ*t*. Based on the spatial steps and time step, the second-order unconditionally stable Crank-Nicolson difference scheme for Eqs. (1) and (4) can be expressed as Eqs. (13) and (14).

13 $$\begin{gathered} \frac{{C_{{s,i}}^{{j+1}} - C_{{s,i}}^{j}}}{{\Delta t}}=\frac{{{D_s}}}{{2R_{{F,s,i}}^{j}}}\left[ {\frac{{C_{{s,i+1}}^{j} - 2C_{{s,i}}^{j}+C_{{s,i - 1}}^{j}}}{{{{\left( {\Delta {x_1}} \right)}^2}}}+\frac{{C_{{s,i+1}}^{{j+1}} - 2C_{{s,i}}^{{j+1}}+C_{{s,i - 1}}^{{j+1}}}}{{{{\left( {\Delta {x_1}} \right)}^2}}}} \right] \hfill \\ - \frac{{{v_s}}}{{2R_{{F,s,i}}^{j}}}\left( {\frac{{C_{{s,i+1}}^{j} - C_{{s,i - 1}}^{j}}}{{2\Delta {x_1}}}+\frac{{C_{{s,i+1}}^{{j+1}} - C_{{s,i - 1}}^{{j+1}}}}{{2\Delta {x_1}}}} \right) - \lambda C_{{s,i}}^{j} \hfill \\ \end{gathered}$$

where $$C_{{s,i}}^{j}={C_s}\left( {{x_i},{t_j}} \right), {x_i}=i \cdot \Delta {x_1}, i=0,1,2, \cdots ,{I_1}, {t_j}=j \cdot \Delta t, j=0,1,2, \cdots ,J.$$

14 $$\begin{gathered} \frac{{C_{{g,i}}^{{j+1}} - C_{{g,i}}^{j}}}{{\Delta t}}=\frac{{{D_g}}}{{2R_{{F,g,i}}^{j}}}\left[ {\frac{{C_{{g,i+1}}^{j} - 2C_{{g,i}}^{j}+C_{{g,i - 1}}^{j}}}{{{{\left( {\Delta {x_2}} \right)}^2}}}+\frac{{C_{{g,i+1}}^{{j+1}} - 2C_{{g,i}}^{{j+1}}+C_{{g,i - 1}}^{{j+1}}}}{{{{\left( {\Delta {x_2}} \right)}^2}}}} \right] \hfill \\ - \frac{{{v_g}}}{{2R_{{F,g,i}}^{j}}}\left( {\frac{{C_{{g,i+1}}^{j} - C_{{g,i - 1}}^{j}}}{{2\Delta {x_2}}}+\frac{{C_{{g,i+1}}^{{j+1}} - C_{{g,i - 1}}^{{j+1}}}}{{2\Delta {x_2}}}} \right) \hfill \\ \end{gathered}$$

where $$C_{{g,i}}^{j}={C_g}\left( {{x_i},{t_j}} \right), {x_i}={I_1}+(i - {I_1})\Delta {x_2}, i={I_1},{I_1}+1,{I_1}+2, \cdots ,{I_1}+{I_2}, {t_j}=j \cdot \Delta t,$$$$j=0,1,2, \cdots ,J.$$.

Moreover, the difference expressions of the Freundlich adsorption retardation factors in the soil-bentonite mixture layer and GCL are obtained using Eqs. (15) and (16).

15 $$R_{F,s,i}^j = 1 + \frac{{{\rho _{d,s}}}}{{{n_s}}}{K_{F,s}}{N_s}C_s^i{,^j}^{{N_s} - 1}$$

16 $$R_{F,g,i}^j = 1 + \frac{{{\rho _{d,g}}}}{{{n_g}}}{K_{F,g}}{N_g}{C_g}{,_i}^j{,^{{N_g} - 1}}$$

Accordingly, the difference formats for the initial, boundary, and continuity conditions are written as Equations (17) to (21).

17 $$C_{{s,i}}^{0}=C_{{g,i}}^{0}=0$$

18 $$C_{{s,0}}^{{j+1}}={C_0}$$

19 $$C_{{s,{I_1}}}^{{j+1}}=C_{{g,{I_1}}}^{{j+1}}$$

20 $$- {n_s}{D_s}\frac{{C_{{s,{I_1}}}^{{j+1}} - C_{{s,{I_1} - 1}}^{{j+1}}}}{{\Delta {x_1}}}= - {n_g}{D_g}\frac{{C_{{g,{I_1}+1}}^{{j+1}} - C_{{g,{I_1}}}^{{j+1}}}}{{\Delta {x_2}}}$$

21 $$\frac{{C_{{g,{I_1}+{I_2}}}^{{j+1}} - C_{{g,{I_1}+{I_2} - 1}}^{{j+1}}}}{{\Delta {x_2}}}= - \mu C_{{g,{I_1}+{I_2}}}^{{j+1}}$$

It should be noted that the Darcy velocity in the soil-bentonite mixture layer is the same as that in the GCL, and the concentration continuity condition is satisfied at the interface of the soil-bentonite mixture layer and GCL. Consequently, the continuity condition for transport flux in Eq. (20) is simplified.

Based on the aforementioned Crank–Nicolson difference expressions for contaminant transport in the soil-bentonite mixture layer and GCL, the contaminant concentration in both the soil-bentonite mixture layer and GCL at an arbitrary time can be obtained through MATLAB programming.

## Validation of the proposed method

**Fig. 2:**
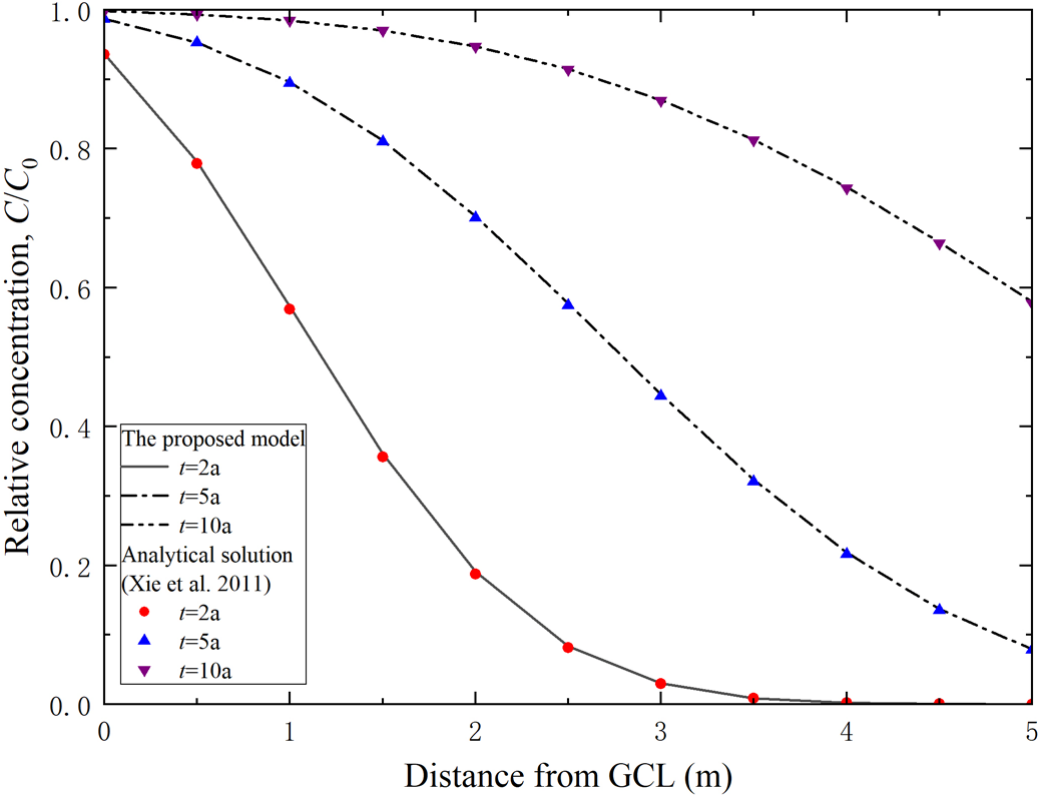
Comparison of contaminant concentration distributions in the composite vertical cut-off wall between the proposed method and existing analytical solutions.

Xie et al.^49^ developed an analytical solution for one-dimensional transport of contaminants through a dual-layer barrier system with GCL, which provides a basis for comparison with the current study. However, the analytical transport model proposed by Xie et al.^49^ did not account for the effects of degradation and assumed a linear adsorption relationship. The proposed method in this study addresses these two factors and provides a more general transport solution of degradable contaminants. Assumption *N* = 1 was employed to simplify the nonlinear adsorption model into a linear adsorption model, the parameter *l* was set to 0 to ignore the degradation of degradable contaminants. Consequently, the governing Eqs. (1) and (4) were modified to align with the conditions applicable to the analytical solution derived by Xie et al.^50^ Furthermore, the initial and boundary conditions were derived from those utilized in the analytical model of Xie et al.^49^. The results obtained by the proposed method and the analytical solution of Xie et al.^49^ are compared in Fig. 2. The concentration distribution curves of contaminants generated by both methods demonstrate strong agreement at *t* = 5, 10 and 20 a, thereby validating the accuracy of the proposed method.

**Fig. 3:**
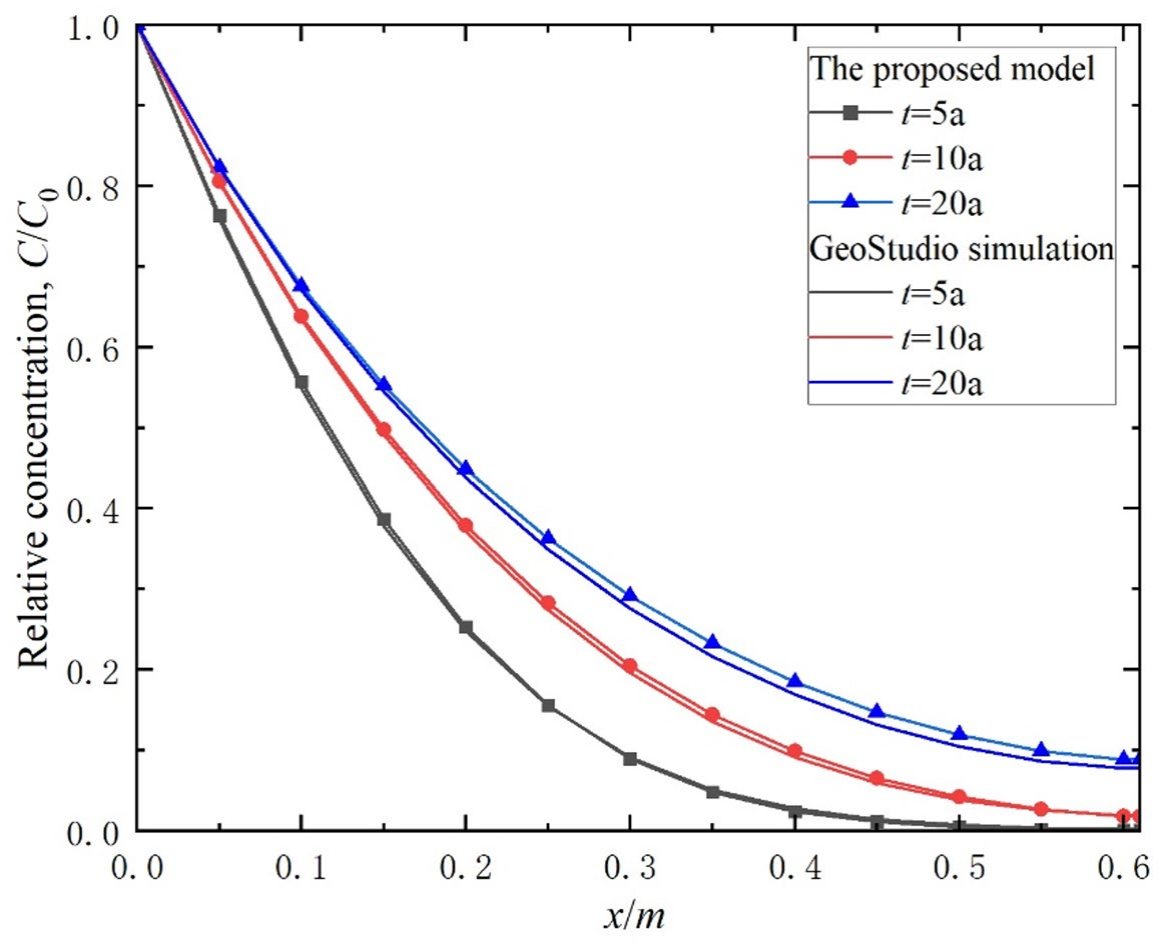
Comparison of concentration distributions between the proposed method and numerical simulations.

To further validate the accuracy of the proposed method, this study compares its calculation results with numerical simulation outcomes obtained using finite element software (GeoStudio version 24.2.0.298). GeoStudio is capable of modeling contaminant transport within geological strata, including the nonlinear adsorption of contaminants within the composite vertical cut-off wall^50^. The parameters outlined in Table 1 were selected for this analysis, and the concentration distribution curves of contaminants generated by both the proposed method and numerical simulations are illustrated in Fig. 3. Notably, the concentration distribution curves from both methods exhibit strong agreement at *t* = 5, 10, and 20 years. Thus, the established theoretical model is applicable for predicting the one-dimensional transport behavior of organic contaminants in the composite vertical cut-off wall.

## Effect of nonlinear adsorption on contaminant transport

For contaminant transport in the composite vertical cut-off wall as shown in Fig. 1, the influence of nonlinear adsorption characteristics has rarely been considered in the existing literature, while most studies rely on the linear adsorption assumption. Therefore, it is necessary to study the impact of nonlinear adsorption characteristics on contaminant transport behavior. According to the author’s preliminary experiments and literature research^51^, one of the most common nonlinear adsorption models (i.e., Freundlich adsorption model) is adopted in this study. The specific expression of the isothermal Freundlich adsorption model is listed as Eq. (22):

22 $$S={K_F}{C^N}$$

where *S* is the adsorption capacity, *C* is the concentration of the contaminant in the leachate, *K*_*F*_ is the Freundlich adsorption constant related to the adsorption capacity of the adsorbent, and *N* is the Freundlich empirical constant representing the intensity of adsorption^52^. The Freundlich adsorption can degenerate to linear adsorption when *N* = 1. In the subsequent analyses, it is assumed that the contaminant source concentration *C*_0_ is 1 mg/L, the hydraulic head difference on both sides of the composite cut-off wall *h*_*d*_ is 1 m, the Robin-type boundary constant *µ* is 0.1, and the degradation half-life is 10 a. Other model parameters are adopted from the literature and shown in Table 1. The breakthrough criterion is set at 0.1, which means that the barrier is considered penetrated when the ratio of the outlet concentration to the source concentration reaches 0.1.

**Table 1:** Parameters for the transport model of degradable contaminants in the composite vertical cut-off wall.

Parameter	Soil-Bentonite Mixture Layer	GCL	References
Thickness, *l* (m)	0.6	0.01	None
Hydraulic conductivity, *k* (m/s)	1.9 × 10^− 10^	5.0 × 10^− 11^	Britton et al.^53^; Xie et al.^21^
Effective diffusion coefficient, D^*^ (m^2^/s)	3.78 × 10^− 10^	3.0 × 10^− 10^	Xie et al.^21^
Longitudinal dispersivity, *α*_*L*_ (m)	0.01	0	Jiang et al.^35^
Porosity, *n*	0.30	0.70	Xie et al.^21^
Freundlich adsorption constant, *K*_*F*_ (L/kg)	0.63	9.60	Li et al.^54^; adsorption tests by the authors (GCL)
Freundlich empirical constant, *N*	0.80	0.98	Li et al.^54^; adsorption tests by the authors (GCL)
Dry density, *ρ*_*d*_ (g/cm^3^)	1.90	0.79	Xie et al.^49^

**Fig. 4:**
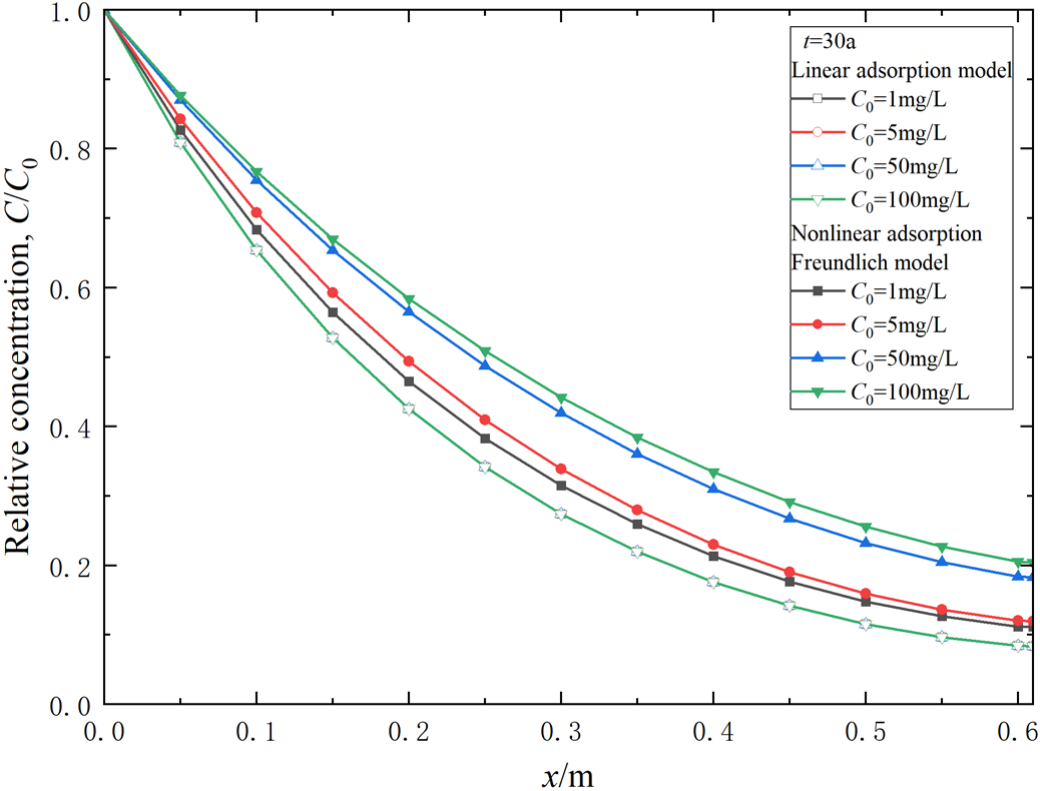
Relative concentration distribution of contaminants considering linear and nonlinear adsorption assumptions (*t* = 30 a).

Figure 4 presents the distribution of relative contaminant concentration in the composite vertical cut-off wall at *t* = 30 a, under two different adsorption conditions. The data indicate that the relative concentration in the composite cut-off wall based on Freundlich adsorption is consistently higher than that derived from the linear adsorption assumption. This discrepancy suggests that the linear adsorption model may underestimate both the range and concentration of contaminants during transport. Consequently, it could result in an overly optimistic estimation of the duration until the contaminant concentration in the effluent surpasses safety regulations, known as the breakthrough time. An increase in the contaminant source concentration (*C*_0_) leads to a greater relative concentration according to the Freundlich adsorption assumption, while the relative concentration based on the linear adsorption model remains unchanged regardless of variations in *C*_0_. The higher the contaminant concentration, the more pronounced the influence of nonlinear adsorption on transport behavior.

**Fig. 5:**
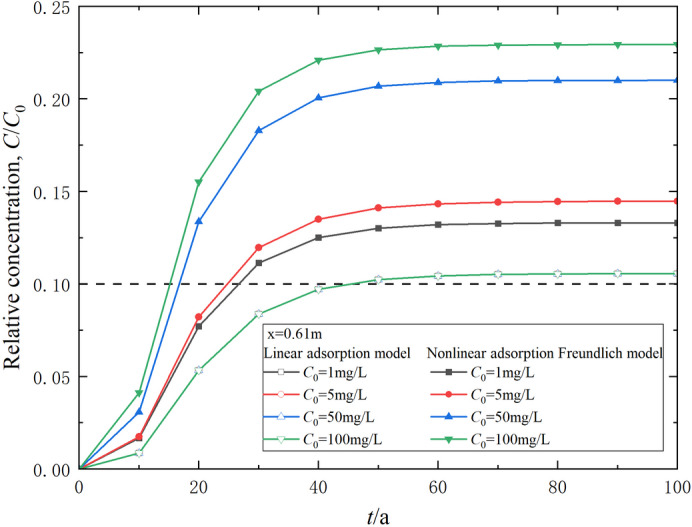
Variation of contaminant relative concentration in effluent over time.

Variation of contaminant relative concentration in effluent behind the composite vertical cut-off wall over time is shown in Fig. 5. It is evident that the contaminant concentration at the outlet boundary of the composite cut-off wall is below the breakthrough criterion of 0.1 at *t* = 30 a under linear assumption, indicating that the breakthrough time of the composite cut-off wall exceeds 30 a. However, the breakthrough time becomes less than 30 a under the Freundlich adsorption assumption, decreasing continuously with the increase of the contaminant source concentration *C*_0_. The main reason for this phenomenon is that the retardation factor decreases with the increase of concentration in the Freundlich adsorption model, whereas it remains constant in the linear adsorption model. Therefore, selecting an appropriate adsorption assumption before calculating the transport law of contaminants in the strata is necessary. From the analysis of Figs. 4 and 5, it can be concluded that contaminant transport analysis based on the linear adsorption assumption can result in significant errors when barrier materials exhibit substantial nonlinear adsorption characteristics toward contaminants. The contaminant transport analysis method proposed in this paper, based on the nonlinear adsorption assumption, has significant practical value for analyzing contamination prevention and control in geology and geo-environmental engineering.

## Parametric studies

In the one-dimensional transport of degradable contaminants within the composite vertical cut-off wall, the nonlinear adsorption constant and the degree of degradation are crucial parameters influencing the transport process^55^. To further elucidate the transport mechanism of degradable contaminants in the composite vertical cut-off wall, a sensitivity analysis of several key model parameters and boundary conditions was conducted. The relative concentration distribution of contaminants and the breakthrough time were calculated to compare the effects of the Freundlich adsorption constant, degradation half-life, and Robin-type boundary constant. In each analysis series, only the variable of interest was modified, while all other parameters were consistent with those presented in Table 1.

### Effect of the Freundlich adsorption constant

**Fig. 6:**
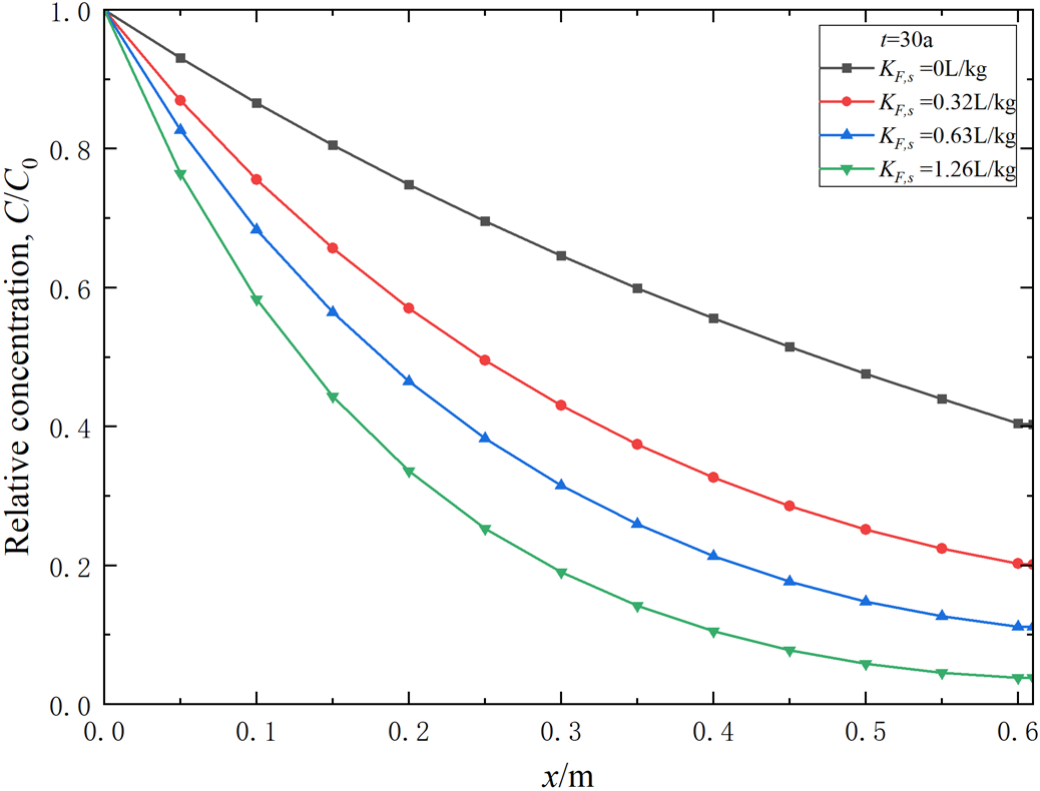
Effect of the Freundlich adsorption constant on the relative concentration distribution of contaminants in the composite vertical cut-off wall (*t* = 30 a).

**Fig. 7:**
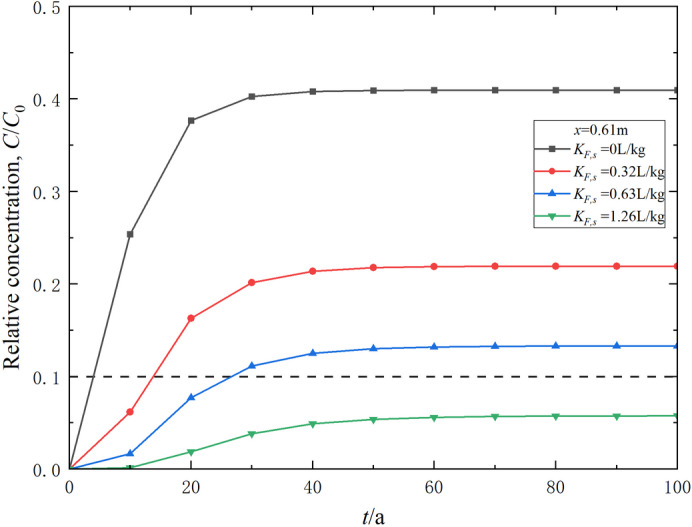
Effect of the Freundlich adsorption constant on the variation of contaminant relative concentration in effluent over time.

The Freundlich adsorption constant *K*_*F*_ generally indicates the strength of the adsorption capacity. By keeping the Freundlich empirical constant *N* constant and varying the Freundlich adsorption constant *K*_*F*,*s*_ of the soil-bentonite mixture layer, the effect of the Freundlich adsorption constant on the transport of contaminants can be explored, as shown in Figs. 6 and 7. Obviously, the Freundlich adsorption constant *K*_*F*_ significantly impacts the contaminant transport process. An increase in *K*_*F*,*s*_ leads to a marked decrease in the contaminant relative concentration at the same position and time (Fig. 6). When *K*_*F*,*s*_ is 0 L/kg, indicating that the adsorption of contaminants on the soil-bentonite mixture layer is neglected, the breakthrough time is about 5.2 a (Fig. 7). When *K*_*F*,*s*_ = 0.32 and 0.63 L/kg, the corresponding breakthrough times are 13.0 and 25.6 a, respectively. As *K*_*F*,*s*_ increases from 0 to 0.32 and 0.63 L/kg, the breakthrough time increases by 150% and 392%, respectively. Thus, barrier materials with greater adsorption capacity certainly exhibit larger breakthrough times. Accordingly, utilizing barrier systems with better adsorption capabilities can effectively delay the breakthrough time of contaminants.

### **Effect of the degradation half-life**

**Fig. 8:**
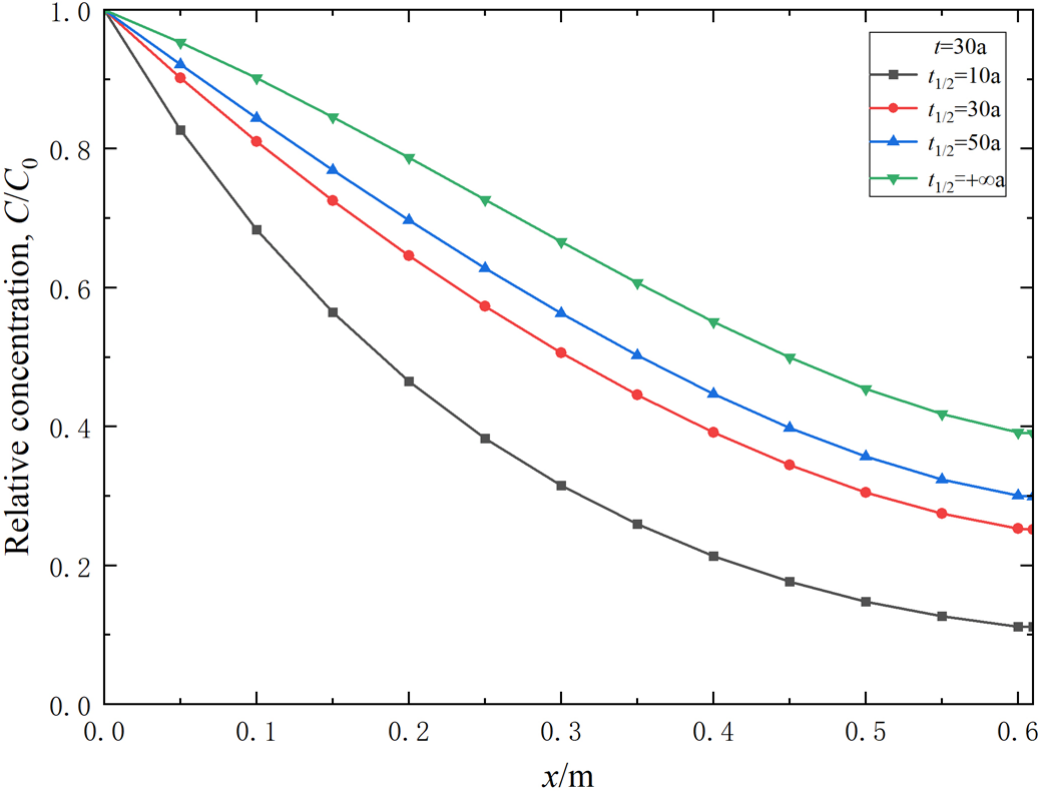
Effect of contaminant degradation half-life on its relative concentration distribution in the composite vertical cut-off wall (*t* = 30 a).

**Fig. 9:**
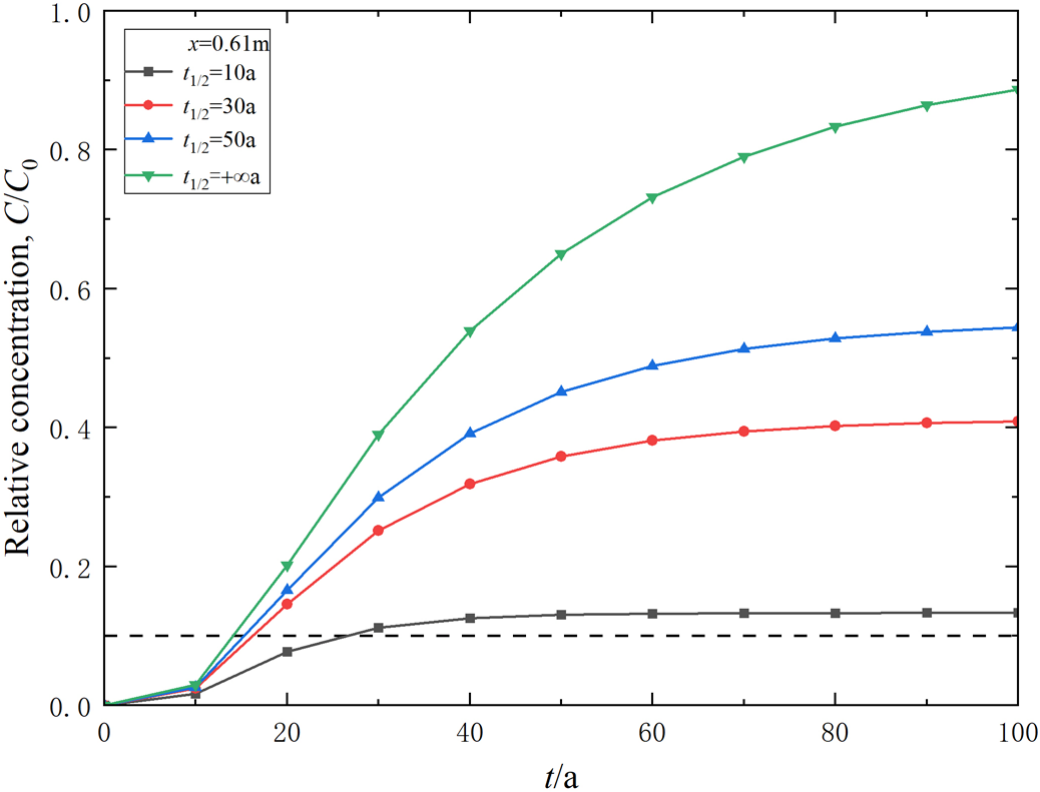
Effect of contaminant degradation half-life on the variation of contaminant relative concentration in effluent over time.

The degradation half-life of degradable contaminants *t*_1/2_ is an important parameter reflecting the rate of degradation. Common organic contaminants such as benzene have a degradation half-life generally ranging from 10 to 50 a^56^. Parametric studies have been performed to study the effect of the degradation half-life on the transport of degradable contaminants. The results are shown in Figs. 8 and 9. It can be observed that the degradation half-life of contaminants *t*_1/2_ notably impacts the transport process of these contaminants. An increase in *t*_1/2_ causes the relative concentration of degradable contaminants to rise at the same position and time (Fig. 8). When *t*_1/2_ = 10, 30, 50 and + ∞ a, the corresponding breakthrough times are 25.6, 17.7, 16.5, and 14.7 a, respectively (Fig. 9). Compared to the case without considering degradation (i.e., *t*_1/2_ = +∞ a), the breakthrough times for *t*_1/2_ = 10 a, *t*_1/2_ = 30 a and *t*_1/2_ = 50 an increase by 74.1%, 20.4%, and 12.2%, respectively. This is mainly due to the longer degradation half-life resulting in a slower degradation rate, which reduces the effect of degradation. Therefore, reducing the degradation half-life of contaminants and enhancing the degradation effect within the composite cut-off wall can improve the impermeability and contamination barrier performance of the wall.

### Effect of the Robin-type constant

**Fig. 10:**
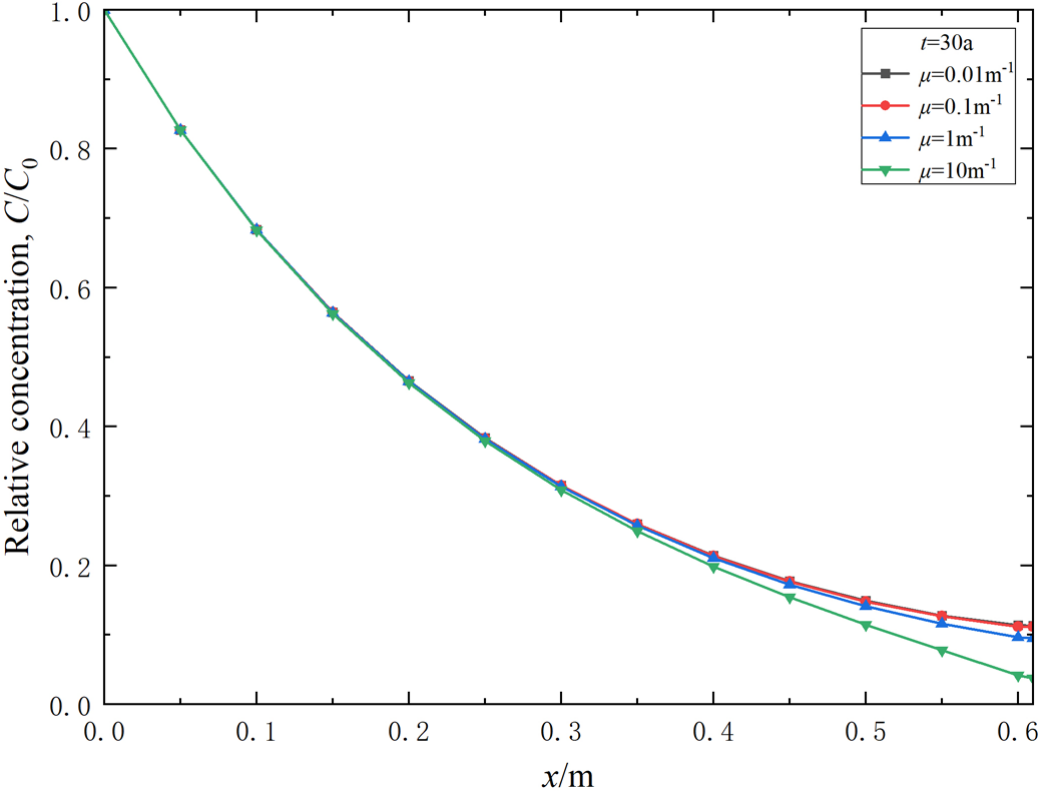
Effect of the Robin-type constant (boundary condition) on the relative concentration distribution of contaminants in the composite vertical cut-off wall (*t* = 30 a).

**Fig. 11:**
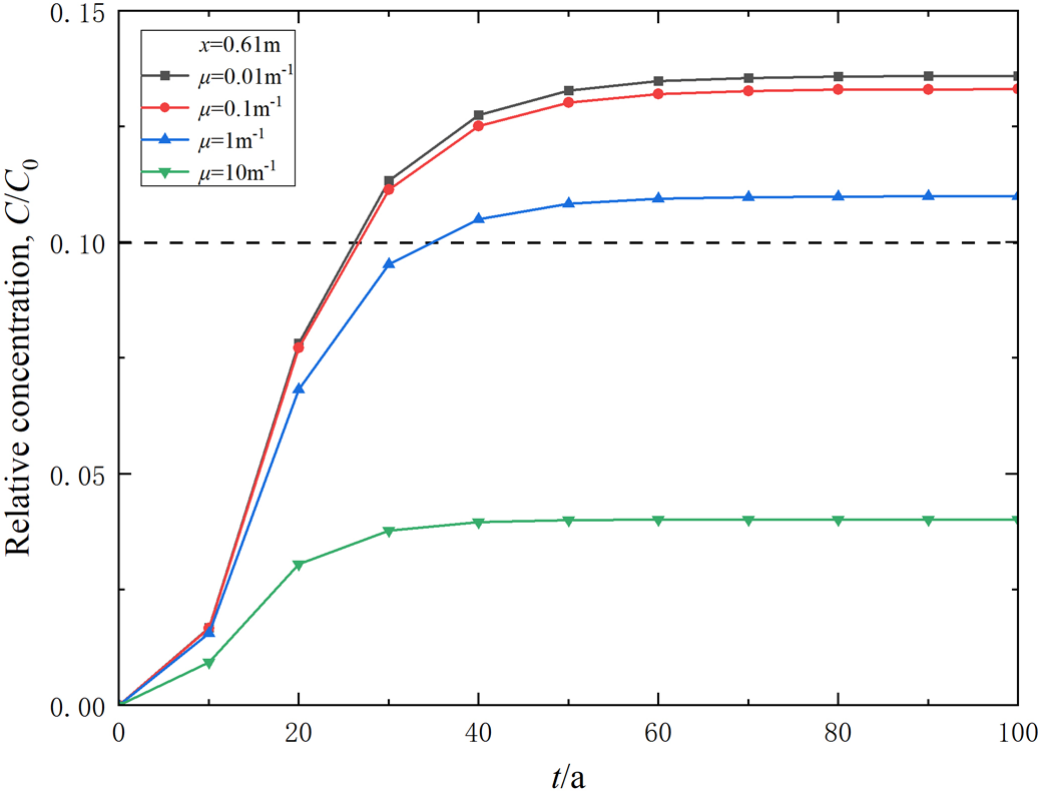
Effect of the Robin-type constant (boundary condition) on the variation of relative concentration of contaminants in effluent over time.

The Robin-type constant *µ* relates to the diffusivity and permeability of outlet boundary conditions. The effect of the Robin-type constant on the transport of contaminants can be illustrated in Figs. 10 and 11. The results indicate that the Robin-type constant *µ* significantly impacts the contaminant relative concentration in the right outlet range of the model. As *µ* increases, the final concentration in the right outlet range of the model decreases (Fig. 10). When *µ* is 0.01, 0.1 and 1 m^− 1^, the corresponding breakthrough times are 25.2, 25.6, and 33.5 a, respectively (Fig. 11). This can be primarily attributed to the fact that as *µ* increases, the permeability of the aquifer layer improves, allowing contaminants migrating near the right outlet of the model to be diluted more rapidly by the flow. Thus, higher permeability of the aquifer in the boundary conditions leads to a corresponding increase in breakthrough time.

## Conclusions

The composite vertical cut-off wall, composed of an adsorptive soil-bentonite mixture and a GCL, exhibits excellent hydraulic conductivity. However, the barrier material within the composite vertical cut-off wall, particularly bentonite, may exhibit pronounced nonlinear adsorption characteristics toward degradable contaminants in leachate. This study establishes a one-dimensional theoretical model for the transport of degradable contaminants in the composite vertical cut-off wall, considering nonlinear adsorption, advection, diffusion, mechanical dispersion, and degradation processes. The finite difference method is employed to numerically solve the theoretical model, and the proposed method is validated through comparative analyses with an existing analytical solution. By comparing and analyzing the relative concentration distribution of degradable contaminants in the composite vertical cut-off wall, as well as the time required for the effluent contaminant concentration to exceed safety regulations (i.e., breakthrough time), the influencing mechanisms governing the transport of degradable contaminants in the composite vertical cut-off wall are elucidated. The main conclusions are as follows:

1. The distribution of relative concentrations of contaminants in the composite vertical cut-off wall under Freundlich nonlinear adsorption conditions is greater than that observed under the linear adsorption assumption. Consequently, the breakthrough time for the composite vertical cut-off wall under Freundlich nonlinear adsorption conditions is shorter than that under the linear adsorption assumption. The *R*_*F*_ under the Freundlich nonlinear adsorption model decreases with increasing contaminant concentration, which primarily accounts for the observed differences in contaminant concentration distribution within the composite vertical cut-off wall compared to linear adsorption.
2. An increase in the Freundlich adsorption constant (*K*_*F*_) slows the transport process of contaminants within the composite vertical cut-off wall. In scenarios where the adsorption of contaminants by barrier materials is disregarded, the breakthrough time for contaminants in the composite barrier wall increases by 346% when the adsorption parameter (*K*_*F*,*s*_ = 0.63 L/kg) is taken into account. Employing barrier materials with strong adsorption capabilities can substantially enhance the breakthrough time of the composite vertical cut-off wall against target contaminants.
3. The degradation process diminishes the relative concentration of contaminants in the composite vertical cut-off wall, and the breakthrough time considering a degradation half-life of contaminants (t_1/2_ = 10 a) is 1.7 times longer than that without accounting for degradable. Additionally, the breakthrough time of the composite vertical cut-off wall against degradable contaminants increases significantly as the degradation half-life of contaminants decreases. Accelerating the degradation of pollutants in soil-bentonite vertical barrier walls can improve the barrier effectiveness of the composite vertical cut-off wall against degradable contaminants.
4. The Robin-type boundary condition constant (*µ*) represents the permeability and diffusivity of the aquifer at the outlet boundary. A larger *µ* signifies improved permeability of the aquifer, resulting in a reduction in contaminant concentration in the effluent leachate on the right side of the model, which corresponds to an increase in breakthrough time. When the outlet boundary is an aquifer with specified permeability, applying the Robin-type boundary for contaminant transport analysis is appropriate for practical applications.

## Data Availability

The datasets used and/or analysed during the current study available from the corresponding author on reasonable request.

## Abbreviations

L_s_: Thicknesses of the soil-bentonite mixture (m)
L_g_: Thicknesses of GCL (m)
L: Thickness of the entire composite vertical cut-off wall (m)
h_d_: Hydraulic head difference (m)
C_0_: Contaminant concentration in the leachate (mg/L)
S: Adsorption capacity (mg/kg)
D_s_, D_g_: Hydrodynamic dispersion coefficients in the soil-bentonite mixture and GCL,:Respectively (m^2^ /s)
v_s_, v_g_: Liquid flow velocities in the soil-bentonite mixture and GCL, respectively (m/s)
R_F,s_, R_F,g_: Retardation factors in the soil-bentonite mixture and GCL, respectively
n_s_, n_g_: Porosities of the soil-bentonite mixture and GCL, respectively
ρ_d,s_, ρ_d,g_: Dry densities of the soil-bentonite mixture and GCL, respectively (g/cm^3^)
K_F,s_, K_F,g_: Freundlich adsorption constants in the soil-bentonite mixture and GCL, respectively (L/kg)
N_s_, N_g_: Freundlich empirical constants in the soil-bentonite mixture and GCL, respectively
λ: Degradation rate coefficient (a^− 1^)
t_1/2_: Degradation half-life (a)
D_s_^*^, D_g_^*^: Effective diffusion coefficients in the soil-bentonite mixture and GCL, respectively (m^2^ /s)
D_m,s_, D_m,g_: Mechanical dispersion coefficients in the soil-bentonite mixture and GCL, respectively (m^2^/s)
α_L,s_, α_L,g_: Longitudinal dispersivities in the soil-bentonite mixture and GCL, respectively (m)
µ: Robin-type boundary constant

## References

[1] Chen, X., Dai, Y., Zhao, M., Löffler, F. E. & Zhuang, J. Hydrobiological mechanism controlling the synergistic effects of unsaturated flow and soil organic matter on the degradation of emerging organic contaminants in soils. *Environ. Sci. Technol.* **56** (16), 11409–11417 (2022). 35905382 10.1021/acs.est.2c03013

[2] Umar, M. et al. Integrating DFT and machine learning for the design and optimization of sodium alginate-based hydrogel adsorbents: efficient removal of pollutants from wastewater. *Environ. Res.* **247**, 118219 (2024). 38253197 10.1016/j.envres.2024.118219

[3] Philip, L. K. An investigation into contaminant transport processes through single-phase cement–bentonite slurry walls. *Eng. Geol.* **60** (1–4), 209–221 (2001).

[4] Zhang, W. B. et al. Compressibility and hydraulic conductivity of sand-attapulgite cut-off wall backfills. *J. Zhejiang Univ. -Sci A*. **20**, 218–228 (2019).

[5] Zhan, L. T., You, Y. Q., Zhao, R., Chen, C. & Chen, Y. M. Centrifuge modelling of lead retardation in soil–bentonite cut-off walls. *Int. J. Phys. Model. Geotech.* **23** (4), 166–179 (2022).

[6] Liu, J. et al. Analytical solution for contaminant transport through a GCL-enhanced composite cutoff wall system. *Comput. Geotech.* **164**, 105828 (2023).

[7] Peng, M. Q. et al. Analytical solution for contaminant transport through a Soil–Bentonite (SB)/Geosynthetic clay liner (GCL)/Soil–Bentonite (SB) composite cutoff wall and an aquifer. *Processes* **12** (7), 1486 (2024).

[8] Mahler, C. F. & Velloso, R. Q. Diffusion and sorption experiments using a DKS permeameter. *Eng. Geol.* **60** (1–4), 173–179 (2001).

[9] Rowe, R. K., Mukunoki, T. & Sangam, H. P. BTEX diffusion and sorption for a geosynthetic clay liner at two temperatures. *J. Geotech. Geoenviron Eng.* **131** (10), 1211–1221 (2005).

[10] Mirbagheri, S. A., Hashemi Monfared, S. A. & Kazemi, H. R. Simulation modelling of pollutant transport from leachate in Shiraz landfill. *Environ. Earth Sci.* **59**, 287–296 (2009).

[11] Shackelford, C. D. The ISSMGE Kerry Rowe lecture: the role of diffusion in environmental geotechnics. *Can. Geotech. J.* **51** (11), 1219–1242 (2014).

[12] Mustafa, S., Bahar, A., Aziz, Z. A. & Suratman, S. Modelling contaminant transport for pumping wells in riverbank filtration systems. *J. Environ. Manage.* **165**, 159–166 (2016). 26433356 10.1016/j.jenvman.2015.09.026

[13] Yadav, R. R. & Kumar, L. K. Analytical solution of two-dimensional Conservative solute transport in a heterogeneous porous medium for varying input point source. *Environ. Earth Sci.* **80**, 1–10 (2021).

[14] Saadatpour, M., Goeini, M., Afshar, A. & Shahmirnoori, A. A preliminary approach based on numerical simulation modelling and evaluation of permeable reactive barrier for aquifer remediation susceptible to selenium contaminant. *J. Environ. Manage.* **331**, 117242 (2023). 36630800 10.1016/j.jenvman.2023.117242

[15] Park, E. & Zhan, H. Analytical solutions of contaminant transport from finite one-, two-, and three-dimensional sources in a finite-thickness aquifer. *J. Contam. Hydrol.* **53** (1–2), 41–61 (2001). 11816994 10.1016/s0169-7722(01)00136-x

[16] Foose, G. J., Benson, C. H. & Edil, T. B. Analytical equations for predicting concentration and mass flux from composite liners. *Geosynth Int.* **8** (6), 551–575 (2001).

[17] Li, Y. C. & Cleall, P. J. Analytical solutions for advective–dispersive solute transport in double-layered finite porous media. *Int. J. Numer. Anal. Methods Geomech.* **35** (4), 438–460 (2011).

[18] Foose, G. J. Transit-time design for diffusion through composite liners. *J. Geotech. Geoenviron Eng.* **128** (7), 590–601 (2002).

[19] Chaudhary, M., Thakur, C. K. & Singh, M. K. Analysis of 1-D pollutant transport in semi-infinite groundwater reservoir. *Environ. Earth Sci.* **79**, 1–23 (2020).

[20] Zhang, Z., Tian, G. & Han, L. Influence of chemical osmosis on solute transport and fluid velocity in clay soils. *Open. Chem.* **18** (1), 232–238 (2020).

[21] Xie, H., Chen, Y. & Lou, Z. An analytical solution to contaminant transport through composite liners with geomembrane defects. *Sci. China Tech. Sci.* **53**, 1424–1433 (2010).

[22] Zhang, W. J. & Qiu, Q. W. Analysis on contaminant migration through vertical barrier walls in a landfill in China. *Environ. Earth Sci.* **61**, 847–852 (2010).

[23] Lin, H., Huang, W., Wang, L. & Liu, Z. Transport of organic contaminants in composite vertical Cut-Off wall with defective HDPE geomembrane. *Polymers* **15** (14), 3031 (2023). 37514421 10.3390/polym15143031 PMC10385536

[24] Peng, C. H. et al. A two-dimensional analytical solution for organic contaminant diffusion through a composite geomembrane cut-off wall and an aquifer. *Comput. Geotech.* **119**, 103361 (2020).

[25] Bouchelaghem, F. & Jozja, N. Multi-scale study of permeability evolution of a bentonite clay owing to pollutant transport: part I. Model derivation. *Eng. Geol.* **108** (1–2), 119–132 (2009).

[26] Chakraborty, R. & Ghosh, A. Three-dimensional analysis of contaminant migration through saturated homogeneous soil media using FDM. *Int. J. Geomech.* **13** (6), 699–712 (2013).

[27] Faisal, A. A. & Abd Ali, Z. T. Groundwater protection from lead contamination using granular dead anaerobic sludge biosorbent as permeable reactive barrier. *Desalin. Water Treat.* **57** (9), 3891–3903 (2016).

[28] Zhang, Z., Masum, S. A., Tian, G. & Thomas, H. R. Modelling non-isothermal volume change and solute transport behaviours of a semi-permeable clay soil under the combined influence of mechanical loading, chemical-osmosis, and thermo-osmosis. *Eng. Geol.* **293**, 106271 (2021).

[29] An, N., Zagorščak, R. & Thomas, H. R. Adsorption characteristics of rocks and soils, and their potential for mitigating the environmental impact of underground coal gasification technology: a review. *J. Environ. Manage.* **305**, 114390 (2022). 34999446 10.1016/j.jenvman.2021.114390

[30] Auton, L. C., Aguareles, M., Valverde, A., Myers, T. G. & Calvo-Schwarzwalder M.An analytical investigation into solute transport and sorption via intra-particle diffusion in the dual-porosity limit. *Appl. Math. Model.* **130**, 827–851 (2024).

[31] Hu, Q., Lan, R., He, L., Liu, H. & Pei, X. A critical review of adsorption isotherm models for aqueous contaminants: curve characteristics, site energy distribution and common controversies. *J. Environ. Manage.* **329**, 117104 (2023). 36603322 10.1016/j.jenvman.2022.117104

[32] Malusis, M. A., Maneval, J. E., Barben, E. J., Shackelford, C. D. & Daniels, E. R. Influence of adsorption on phenol transport through soil–bentonite vertical barriers amended with activated carbon. *J. Contam. Hydrol.* **116** (1–4), 58–72 (2010). 20609493 10.1016/j.jconhyd.2010.06.001

[33] Wang, Y., Chen, Y., Xie, H., Zhang, C. & Zhan, L. Lead adsorption and transport in loess-amended soil-bentonite cut-off wall. *Eng. Geol.* **215**, 69–80 (2016).

[34] Tian, C. et al. Study on cadmium retardation by Guar gum modified bentonite barrier wall. *Desalin. Water Treat.* **317**, 100212 (2024).

[35] Jiang, W., Ge, S., Feng, C. & Li, J. Transport of heavy metal contaminants in a composite liner under non-isothermal condition. *Geosynth Int.* **31**(4), 1–18 (2023).

[36] Goyal, A., Sanghi, R., Misra, A. K. & Shukla, J. B. Modeling and analysis of the removal of an organic pollutant from a water body using fungi. *Appl. Math. Model.* **38** (19–20), 4863–4871 (2014).

[37] Wang, T. et al. Interactions between microplastics and organic pollutants: effects on toxicity, bioaccumulation, degradation, and transport. *Sci. Total Environ.* **748**, 142427 (2020). 33113705 10.1016/j.scitotenv.2020.142427

[38] Alessandrino, L. Effect of Biochar application on physiochemical properties and nitrate degradation rate in a siliciclastic riverine sandy soil. *Environ. Earth Sci.* **83** (19), 573 (2024).

[39] Sleight, T. W., Khanna, V., Gilbertson, L. M. & Ng, C. A. Network analysis for prioritizing biodegradation metabolites of polycyclic aromatic hydrocarbons. *Environ. Sci. Technol.* **54** (17), 10735–10744 (2020). 32692172 10.1021/acs.est.0c02217

[40] Jiang, W., Ge, S. & Li, J. One-dimensional non-isothermal diffusion model for organic pollutant in an unsaturated composite liner considering the degradation effect. *Comput. Geotech.* **176**, 106807 (2024).

[41] Wu, X., Shi, J. & He, J. Analytical solutions for diffusion of organic contaminant through GCL triple-layer composite liner considering degradation in liner. *Environ. Earth Sci.* **75**, 1–18 (2016).

[42] Pu, H., Qiu, J., Zhang, R. & Zheng, J. Analytical solutions for organic contaminant diffusion in triple-layer composite liner system considering the effect of degradation. *Acta Geotech.* **15**, 907–921 (2020).

[43] Muñoz-Vega, E. et al. Role of soil biofilms in clogging and fate of pharmaceuticals: A laboratory-scale column experiment. *Environ. Sci. Technol.* **57** (33), 12398–12410 (2023). 37558209 10.1021/acs.est.3c02034 PMC10448752

[44] Rowe, R. K. & Brachman, R. W. I. Assessment of equivalence of composite liners. *Geosynth Int.* **11** (4), 273–286 (2004).

[45] Ding, X. H., Feng, S. J., Zheng, Q. T. & Peng, C. H. A two-dimensional analytical model for organic contaminants transport in a transition layer-cutoff wall-aquifer system. *Comput. Geotech.* **128**, 103816 (2020).

[46] Peng, M. Q., Feng, S. J., Chen, H. X., Chen, Z. L. & Xie, H. J. Analytical model for organic contaminant transport through GMB/CCL composite liner with finite thickness considering adsorption, diffusion and thermodiffusion. *Waste Manage.* **120**, 448–458 (2021). 10.1016/j.wasman.2020.10.004 33139192

[47] Feng, S. J., Peng, M. Q., Chen, H. X. & Chen, Z. L. Fully transient analytical solution for degradable organic contaminant transport through GMB/GCL/AL composite liners. *Geotext. Geomembr.* **47** (3), 282–294 (2019).

[48] Alshawabkeh, A. N. & Rahbar, N. Parametric study of one-dimensional solute transport in deformable porous media. *J. Geotech. Geoenviron Eng.* **132** (8), 1001–1010 (2006).

[49] Xie, H., Lou, Z., Chen, Y., Jin, A. & Chen, P. An analytical solution to contaminant advection and dispersion through a GCL/AL liner system. *Chin. Sci. Bull.* **56**, 811–818 (2011).

[50] Chen, H. et al. Study on Rule of Heavy Metal Cu2 + in Small and Medium-Sized Tailings Ponds under Rainfall-Evaporation-Transpiration Coupling. In IOP Conference Series: Earth and Environmental Science.668(1) (2021).

[51] Suzuki, M. *Adsorption engineering. Chem. Eng. Monogr* Vol. 25 (Kodansha, 1990).

[52] Yang, C. H. Statistical mechanical study on the Freundlich isotherm equation. *J. Colloid Interface Sci.* **208** (2), 379–387 (1998). 9845681 10.1006/jcis.1998.5843

[53] Britton, J. P., Filz, G. M. & Little, J. C. The effect of variability in hydraulic conductivity on contaminant transport through soil–bentonite cutoff walls. *J. Geotech. Geoenviron Eng.* **131** (8), 951–957 (2005).

[54] Li, J. S., Jiang, W. H., Ge, S. Q. & H, X. Study on the coupling model for consolidation and contaminant transport of compacted clay liner under non-isothermal distribution condition. *Chin. J. Geotech. Eng.* **44** (11), 2071–2080 (2022).

[55] Zhan, T. L., Zhan, X., Lin, W., Luo, X. & Chen, Y. Field and laboratory investigation on geotechnical properties of sewage sludge disposed in a pit at Changan landfill, Chengdu, China. *Eng. Geol.* **170**, 24–32 (2014).

[56] Xie, H. et al. An analytical solution to organic contaminant diffusion through composite liners considering the effect of degradation. *Geotext. Geomembr.* **36**, 10–18 (2013).

